# An evaluation of the effect of an educational intervention for Australian social workers on competence in delivering brief cognitive behavioural strategies: A randomised controlled trial

**DOI:** 10.1186/1472-6963-10-304

**Published:** 2010-11-05

**Authors:** G Armstrong, G Blashki, L Joubert, R Bland, R Moulding, J Gunn, L Naccarella

**Affiliations:** 1 Nossal Institute for Global Health, The University of Melbourne, Carlton, Victoria, Australia; 2 Department of Social Work, The University of Melbourne, Carlton, Victoria, Australia; 3 School of Social Work & Human Services, The University of Queensland, St Lucia, Queensland, Australia; 4 Swinburne Psychological Clinical Health and Evaluation Research Centre, Swinburne University of Technology, Hawthorn, Victoria, Australia; 5 Department of General Practice, The University of Melbourne, Carlton, Victoria, Australia; 6 Australian Health Workforce Institute, The University of Melbourne, Carlton, Victoria, Australia

## Abstract

**Background:**

Broad community access to high quality evidence-based primary mental health care is an ongoing challenge around the world. In Australia one approach has been to broaden access to care by funding psychologists and other allied health care professionals to deliver brief psychological treatments to general practitioners' patients. To date, there has been a scarcity of studies assessing the efficacy of social worker delivered psychological strategies. This study aims to build the evidence base by evaluating the impact of a brief educational intervention on social workers' competence in delivering cognitive behavioural strategies (strategies derived from cognitive behavioural therapy).

**Methods:**

A randomised controlled trial design was undertaken with baseline and one-week follow-up measurement of both objective and self-perceived competence. Simulated consultations with standardised depressed patients were recorded on videotape and objective competence was assessed by blinded reviewers using the Cognitive Therapy Scale. Questionnaires completed by participants were used to measure self-perceived competence. The training intervention was a 15 hour face-to-face course involving presentations, video example consultations, written materials and rehearsal of skills in pairs.

**Results:**

40 Melbourne-based (Australia) social workers enrolled and were randomised and 9 of these withdrew from the study before the pre training simulated consultation. 30 of the remaining 31 social workers (97%) completed all phases of the intervention and evaluation protocol (16 from intervention and 14 from control group). The intervention group showed significantly greater improvements than the control group in objective competence (mean improvement of 14.2 (7.38-21.02) on the 66 point Cognitive Therapy Scale) and in subjective confidence (mean improvement of 1.28 (0.84-1.72) on a 5 point Likert scale). On average, the intervention group improved from below to above the base competency threshold on the Cognitive Therapy Scale whilst the control group remained below.

**Conclusions:**

Social workers can attain significant improvements in competency in delivering cognitive behavioural strategies from undertaking brief face to face training. This is relevant in the context of health reforms that involve social worker delivery of evidence based psychological care. Further research is required to assess how these improvements in competence translate into performance in practice and clinical outcomes for patients.

## Background

Mental health problems such as depression and anxiety are common and are increasingly recognised as a major contributor to the global health burden [1-4]. There is good evidence that psychological treatments such as cognitive behavioural therapy can be effective in helping recovery and preventing relapse when provided by clinical psychologists [5-7].

An ongoing challenge is to provide broad community access to evidence-based psychological treatments that are high quality, affordable and equitably distributed, and where the intensity of treatments is matched to people's mental health needs [8-11]. Not all services can be provided by specialists, and there is a growing interest in teaching simplified psychological strategies rather than complete therapies [8,12]. With suitable evidence-based training, social workers and other allied health professionals may provide an effective option for non-specialist delivery of brief psychological interventions within the primary care setting.

In Australia, social workers comprise a large and integral proportion of the mental health workforce [13]. Social workers bring to their direct practice an invaluable understanding of how the social environment shapes a person's experience of mental illness, and how that illness impacts on the individual, family, and community [14]. Historically however, evidence-based clinical mental health skills have not been given the same attention in undergraduate and post-graduate social work education, often leaving graduates without the practical skills required in mental health social work practice. This issue is important given the emerging social work opportunities in primary mental health care [15].

In a similar vein to the UK policy of training primary mental health care workers (PMHCWs) in brief therapy techniques [16], there have been major reforms in Australia that fund appropriately qualified social workers and other allied health professionals to provide "focused psychological strategies" derived from evidence based psychological therapies, primarily cognitive behavioural therapy (CBT) [17,18]. It is recognised that focused psychological strategies do not represent CBT in its entirety. Rather, these strategies comprise individual elements of the CBT approach such as structured problem solving, activity planning and sleep wake cycle management, which are selected to allow the delivery of brief psychological interventions in primary care [19-22].

So far, evidence from randomised controlled trials indicates that brief CBT of up to 12 sessions delivered by suitably trained psychologists may be cost-effective, and enable patients with depressive symptoms to recover faster when compared to usual care in general practice [23,24]. However, a systematic review of the literature found that given the lack of relevant studies, it is difficult to determine the efficacy of social worker delivered CBT for depression and anxiety [25].

In response to this gap, we developed a new training programme in focused psychological strategies for social workers (SW-fps). The training programme design was influenced by the increasingly evidence-based approach to mental health training for health professionals [26-28], incorporating a focus on interactive training involving role plays, rehearsal of skills, and the provision of relevant worksheet materials [29]. A randomised controlled trial was undertaken to evaluate the training intervention with baseline and follow up assessments of self-perceived and objectively-measured competence. The central hypothesis was that social workers receiving the training for skills in focused psychological strategies would show greater improvements in both objective and self-perceived competence in delivering cognitive behavioural strategies than untrained social workers.

## Methods

### Participants

Participants were social workers based in the State of Victoria, Australia. Participants were required to have a Bachelor of Social Work degree, at least one year of experience in direct social work practice, and an interest in mental health training. Ethics approval was obtained from the University of Melbourne Health Sciences Human Ethics Sub-Committee. The trial was registered with the International Register of Randomised Controlled Trials prior to commencement (ISRCTN64702482).

### Intervention

The intervention was a multifaceted education programme delivered for five hours per week over three weeks. Three trainers jointly delivered all sessions of the training; 1) an Associate Professor/general practitioner with a doctorate in mental health and extensive experience in delivering CBT training, 2) an Associate Professor with qualifications in both social work and clinical psychology, and 3) a social work researcher with experience in community mental health.

The training aimed to provide social workers with evidence-based cognitive behavioural strategies that are adapted for brief psychological interventions in the primary mental health care setting. These psychological strategies are individual elements of the CBT approach and do not comprise a full course on CBT. The two central components of the training were; 1) the context and theory around focused psychological strategies and mental health social work practice in primary care, and 2) cognitive behavioural strategies for brief psychological interventions with people experiencing high prevalence disorders such as depression and anxiety (see Table 1). A key aspect of the training was the development of practical step-by-step worksheets that social workers could use with clients, particularly as homework tools. A novel aspect of the training was that it integrated eco-mapping[30], a social work psychosocial assessment tool, as a process of locating the client and their presenting issue(s) (e.g. social withdrawal) within their contextual environment. Eco-mapping involves graphically mapping the interactive networks within which the client is embedded.

**Table 1:** Content of educational intervention for social workers in focused psychological strategies (SW-fps)

Contextual and theoretical
➢ Primary mental health care and the role for social work

➢ Introducing focused psychological strategies

➢ The basic theory behind cognitive behavioural strategies

➢ The logistics of working under the new primary mental health care initiatives

**Clinical skills - brief psychological strategies**

➢ Eco-mapping as a social work assessment tool

➢ Structured problem solving

➢ Activity planning

➢ Sleep wake cycle

➢ Slow breathing

➢ Using a cognitive behavioural therapy worksheet in practice

The training sessions were conducted by mental health practitioners who have experience in researching and facilitating mental health training. The training used a combination of learning formats; lectures, role plays, video and group discussion. A training DVD (which included expert interviews and brief role play examples) and workbook were specifically developed and produced for the study.

### Measures

#### Objective competence

The primary outcome measure was the development of objective competence in clinical skills in applying cognitive behavioural strategies. The primary outcome was measured at baseline and one week follow-up using videotaped standardised simulated consultations before and after training, which were each subsequently rated by two blinded reviewers using the Cognitive Therapy Scale (CTS) [31-34]. The CTS is a well-validated 11-item rating scale, with each item having a maximum score of 6, giving an overall maximum possible score of 66. The CTS can be used to rate audio or video-tapes of therapy sessions (or live sessions), and is a measure used in psychotherapy research and training programs. It is divided into two sections; (1) General Therapeutic Skills (6-item scale with a maximum possible score of 36), and (2) Conceptualisation, Strategy, and Technique (5-item scale with a maximum possible score of 30). A score of 39 out of 66 on the overall CTS is considered the threshold for base competency [35].

Three experienced actors from the Medical Education Unit at The University of Melbourne were trained to simulate a 35-year-old woman with mild depression experiencing a strong sense of being overwhelmed by a range of psychosocial problems (e.g., care of elderly parent, financial stress, social isolation). Participants were provided with brief written background information on the client. Participants were instructed to assume it was their third consultation with the client, and that in this twenty minute session their primary task was to assist the client with problem solving. An unattended video camera recorded the consultation.

#### Self Perceived Competency

A questionnaire was developed for the social worker participants to rate their confidence in applying cognitive behavioural strategies using a series of Likert scales (from 0 - not at all confident to 4 - extremely confident) pertaining directly to the content of the training.

### Sample size

Based on our primary outcome measure, we estimated that we would require a total of 40 social workers (20 in each group) to show a difference of 8 points in the mean competency score on the 66 point CTS between the control group and the intervention group at follow-up, with a power of 80% and significance level at 5% for a two-sided test. We made two assumptions about our primary outcome measure based on the results of our previous study applying a training package on focused psychological strategies for general practitioners (GPs) [12]; (1) we assumed a standard deviation of 9, (2) we expected a pre-training starting score on the CTS of approximately 33.

### Randomisation and blinding

The forty consenting participants were randomly allocated into two groups of equal size. Participants were assigned a number and then as each number was randomly drawn from a hat participants were allocated alternating between the intervention and control. Participants and training facilitators were not blinded to the group assignment for pragmatic reasons. The two independent raters who were scoring the social worker participants based on their video-taped simulated consultations were blinded to whether participants were in the control group or the intervention group, and whether it was the participant's baseline or follow-up video-taped simulated consultation. Each simulated consultation video had a unique code identification known only to the primary researcher (GA).

### Analysis

Statistical analysis was performed with SPSS version 18. The internal consistency of the CTS was estimated using Cronbach's Alpha, and inter-rater agreement was estimated using the Pearson product-moment correlation coefficient (Pearson's *r*).

For each social worker, mean clinical competency scores were calculated using the CTS scores from the two raters and analysed using descriptive statistics and t-tests to compare the baseline and follow-up scores between the intervention and control groups. Multiple linear regression analysis was used to calculate the difference in competency scores at follow-up between the study groups. The dependent variable was competency scores at follow-up and the two independent variables were study group and competency scores at baseline to ensure that any differences between the study groups at follow-up was corrected for any differences between individuals (and hence the study groups) at baseline.

Mean self-rated confidence in using cognitive behavioural strategies was calculated for each strategy covered in the training, and an overall mean was calculated across all the strategies. Similarly to the above, regression analysis was used to calculate the differences between study groups in self-reported confidence, adjusting for baseline scores.

Results are reported as differences in means between intervention and control groups for objective competence and self-perceived competence, together with the respective 95% confidence intervals and two-sided p-values. In the results, we also comment on whether with the training, the social worker participants were able to achieve the base objective competency score of 39 on the Cognitive Therapy Scale.

## Results

### Participants and compliance

An email advertisement distributed to 1529 social workers through the Victorian Branch of the Australian Association of Social Workers resulted in 95 expressions of interest. The first 40 respondents to return the signed consent form were consented into the study. Attrition after notification of study status and the dates of the training intervention left 31 participants. One participant from the intervention group was lost to follow-up after receipt of the full training intervention due to illness, leaving a total of 30 participants for analysis; 16 in the intervention group and 14 in the control group.

Table 2 presents the characteristics of the two study groups. The sample was strongly represented by female social workers (80%), with a mean age of 44 and a high level of experience (the mean years of practice was 12.4). A high percentage of participants had previously received training in psychosocial assessment (73.3%) and half (53.3%) had received training in structured problem solving; approximately one-third had received training in slow breathing and activity planning. Almost half the sample worked in sections of the acute health sector that were not mental-health specific.

**Table 2:** Characteristics of the social worker participants by study group; numbers, percentages and averages

Characteristics	Intervention (n = 16)	Control (n = 14)	Total (n = 30)
	
	n (%)	n (%)	n (%)
**Sex**			

Male	5 (31.2)	1 (7.1)	6 (20)

Female	11 (68.8)	13 (92.9)	24 (80)

**Post-graduate qualifications in social work**			

Yes	3 (18.8)	2 (14.3)	5 (16.7)

No	13 (81.2)	12 (85.7)	25 (83.3)

**Member of Australian Association of Social Workers**			

Yes	12 (75)	9 (64.3)	21 (70)

No	4 (25)	5 (35.7)	9 (30)

**Accredited mental health social worker**			

Yes	4 (25)	2 (14.3)	6 (20)

No	12 (75)	12 (85.7)	24 (80)

**Sector of current main employment**			

Community mental health	2 (12.5)	3 (21.4)	5 (16.7)

Community health	2 (12.5)	1 (7.1)	3 (10)

Other community/NGO	0 (0)	2 (14.3)	2 (6.7)

Acute mental health	1 (6.2)	0 (0)	1 (3.3)

Health/hospital (other than acute mental health)	7 (43.8)	6 (42.9)	13 (43.3)

Private practice	2 (12.5)	2 (14.3)	4 (13.3)

Other	2 (12.5)	0 (0)	2 (6.7)

Received previous training in cognitive behavioural strategies*			

Basic CBT techniques	7 (43.7)	6 (42.9)	13 (43.3)

Structured problem solving	10 (62.5)	6 (42.9)	16 (53.3)

Slow breathing	7 (43.7)	4 (28.6)	11 (36.7)

Activity planning	5 (31.2)	5 (35.7)	10 (33.3)

Psychosocial assessment	11 (68.7)	11 (78.6)	22 (73.3)

Sleep wake cycle management	1 (6.3)	1 (7.1)	2 (6.7)

* Participants were asked if they had previously received any training (a seminar/workshop or university studies) in cognitive behavioural strategies

There were moderate differences between the study groups based on the proportion of male social workers and the mean age (40.5 in the intervention group and 47.5 in the control group), though testing revealed no significant differences in age (independent sample *t*(28) = 1.712, *p *= 0.098) and gender (*p *= 0.175, Fisher's exact test) between the study groups.

### Measures

The CTS showed satisfactory internal consistency; the Cronbach alpha coefficient was 0.88 at baseline and 0.94 at follow-up. Satisfactory inter-rater agreement was achieved on the CTS scale at both baseline (*r*(28) = 0.481, *p *= 0.007) and follow-up (*r*(28) = 0.603, *p *< 0.001).

### Effect of the intervention

#### Objective Competence

At baseline, a higher level of objective competence was demonstrated within the intervention group (mean = 28.34) than within the control group (mean = 26.36) although this difference was not statistically significant; two sample *t*(28) = 0.66, *p *= 0.520.

**Figure 1:**
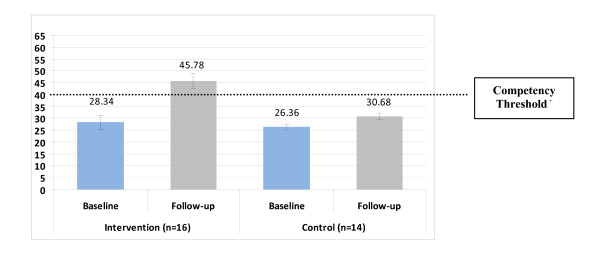
Change in mean competence* from baseline to follow-up by group (error bars indicate standard error). * Measured by video-taped simulated consultations rated by blinded reviewers using the Cognitive Therapy Scale; 11-item scale with a maximum possible score of 66. ^+ ^A score of 39 out of a possible 66 on the Cognitive Therapy Scale is considered the base competency threshold

On average, participants in both study groups displayed increased objective competence between baseline and follow-up, as illustrated by Figure 1. Within the intervention group mean competence ratings on the CTS significantly increased by 17.44 points; paired *t*(15) = 5.30, *p *< 0.001. Within the control group mean competence ratings significantly increased by 4.32 points; paired *t*(13) = 3.83, *p *= 0.002). The mean competence of 45.78 within the intervention group at follow-up was statistically significantly larger than the CTS base competency threshold of 39; one sample *t*(15) = 2.20, *p *= 0.044. The mean competence of 30.68 within the control group at follow-up was statistically significantly smaller than the CTS base competency threshold of 39; one sample *t*(13) = 6.06, *p *< 0.001.

Multiple linear regression analysis was performed (Table 3) to calculate the mean difference in competence at follow-up between the intervention and control groups, adjusting for baseline competence scores. On average, participants in the intervention group were rated 14.20 points higher than their control group counterparts at follow-up when controlling for baseline competency scores, a significant difference. The overall difference between the study groups in competence at follow-up was also statistically significant in each of the two competency subscales, when controlling for baseline competency scores.

**Table 3:** Multiple linear regression analysis of the difference in scores on objective competence and self-perceived competence at follow-up between the intervention and control groups, adjusted for baseline competence

	Baseline ratings	Difference between groups at follow-up	
	**Mean** **(95% C.I)**	**Mean** **(95% C.I)**	**p-value**

Objective Competence*			

Overall cognitive therapy skills			
Control	26.36 (23.81, 28.91)	14.20 (7.38, 21.02)	<0.001
Intervention	28.34 (22.39, 34.30)		

***Subscales***			

General Therapeutic Skills			
Control	15.57 (13.34, 17.80)	7.86 (3.81, 11.90)	<0.001
Intervention	16.56 (13.46, 19.66)		

Conceptualisation, Strategy and Technique			
Control	10.79 (9.58, 11.99)	6.34 (2.73, 9.96)	<0.001
Intervention	11.78 (8.77, 14.79)		

**Self-perceived Competence**^+^			

Overall mean confidence across all strategies			
Control	1.27 (0.94, 1.61)	1.28 (0.84, 1.72)	<0.001
Intervention	1.69 (1.26, 2.11)		

***Individual skill areas***			

Basic overall skills in CBT			
Control	1.07 (0.54, 1.60)	1.59 (1.05, 2.13)	<0.001
Intervention	1.44 (0.85, 2.02)		

Structured problem solving			
Control	1.43 (0.99, 1.87)	1.49 (0.87, 2.12)	<0.001
Intervention	1.88 (1.23, 2.52)		

Slow breathing			
Control	1.14 (0.59, 1.69)	1.16 (0.65, 1.68)	<0.001
Intervention	1.69 (1.05, 2.32)		

Activity planning			
Control	1.21 (0.81, 1.62)	1.37 (0.76, 1.98)	<0.001
Intervention	1.63 (1.01, 2.24)		

Psychosocial assessment			
Control	2.29 (1.63, 2.94)	0.84 (0.29, 1.39)	0.004
Intervention	2.75 (2.18, 3.32)		

Sleep wake cycle management			
Control	0.50 (0.61, 0.94)	1.68 (1.09, 2.28)	<0.001
Intervention	0.75 (0.39, 1.11)		

* Video-taped standardised simulated consultations rated by blinded reviewers using the 66-item Cognitive Therapy Scale

^+ ^Self-reported level of confidence in using cognitive behavioural strategies; Likert scales from 0 (not at all confident) to 4 (extremely confident).

#### Self perceived competence

At baseline, a higher level of self-reported confidence was demonstrated within the intervention group (mean = 1.69) than within the control group (mean = 1.27) although this difference was not statistically significant; two sample *t*(28) = 1.60, *p *= 0.120.

Within the intervention group, the mean confidence across all the focused psychological strategies increased by 1.38, and this difference was significant; paired *t*(15) = 7.73, *p *< 0.001. Within the control group, the mean confidence across all the focused psychological strategies increased by 0.24, and this increase in mean was not significant; paired *t*(13) = 2.11, *p *= 0.055.

Within both study groups, the highest level of self-reported confidence at baseline was observed in the skill area of psychosocial assessment, and the lowest level of baseline confidence was reported in the skill area of sleep-wake cycle management.

Multiple linear regression analysis was performed (Table 3) to calculate the mean differences in self-reported confidence ratings between the intervention and control groups, adjusting for baseline confidence ratings. On average, at follow-up participants in the intervention group rated their overall confidence in using cognitive behavioural strategies 1.28 points higher than the control group on the five-point Likert scales, when controlling for baseline confidence ratings. Mean confidence at follow-up in *each *of the individual cognitive behavioural strategies was significantly higher in the intervention group compared to the control group, when controlling for baseline confidence ratings.

## Discussion

We set out to evaluate an educational intervention for social workers in cognitive behavioural strategies using a randomised controlled trial design. The intervention group showed substantial improvements in both objective and self-perceived competence in a range of cognitive behavioural therapy skills. The control group had a small but statistically significant improvement in objective competence between baseline and follow-up which can largely be explained by a testing effect, whereby the participants performed better due to repeating the same testing measure.

On average, the objective competence within the intervention group improved from below to above the base competency threshold on the Cognitive Therapy Scale whilst the control group remained below. Importantly, competence on the CTS has been demonstrated to be associated with positive patient outcomes when the therapists are trained clinical psychologists [36,37]. While this study does not provide evidence that a brief training intervention can turn social workers into competent cognitive therapists, the findings do provide preliminary evidence that social workers can be trained to competently deliver targeted cognitive behavioural strategies.

The social workers' baseline competency scores were lower than anticipated. During the design of the study we had based our assumption about baseline competency scores on a similar study involving general practitioners in Australia [12]. This may reflect the highly selected nature of those general practitioners who were training to be recognised as level 2 mental health accredited general practioners in the Australian system.

The study was undertaken in the context of major mental health reforms within Australia[18,38] and the UK[16] which have provided funding for health workers, including social workers in Australia, to deliver brief evidence-based psychological therapies. However, a systematic review of the literature had found that given the lack of relevant studies, it is difficult to determine the efficacy of social worker delivered CBT for depression and anxiety [25].

This study provided evidence that a brief educational intervention can prepare social workers to competently deliver targeted cognitive behavioural strategies. Notably, the training intervention encompassed specific elements of the broader CBT approach termed focused psychological strategies, delivered with a strong emphasis on role play and rehearsal of skills, and incorporated an eco-systemic perspective [30] to psychosocial assessment. While we did not perform a formal cost benefit or cost comparison analysis - the brief nature of training required to elevate the skills of the social workers indicates that such an approach may be a cost-effective manner to increase the number of mental health workers available in the general health workforce. In future studies, we wish to examine such issues formally (e.g. effectiveness and cost-effectiveness of therapy following measurement of patient outcomes).

Caution is required in generalising the findings of this study to all social workers. An important limitation of the study was the self selection of a relatively experienced cohort of social workers who had a particular interest in mental health. It is not clear if the same results would be achieved with a different sample, for example, a less experienced cohort of social workers. Additionally, the final sample of thirty social workers is a relatively small sample from which to draw broad generalisations regarding the capacity of social workers. However, despite the small sample, the effect size was sufficiently large to obtain a statistically significant increase in both competence and confidence.

A further limitation of this study was that the training covered a suite of practical psychological strategies and yet in the simulated consultations the social workers were instructed that the primary task was to assist the client with problem solving. Therefore although the trained social workers were able to more competently deliver the one strategy that we selected for the outcome measure, it's possible that they would not have performed as well in other specific skills. Notably there was also a significant increase in self-perceived competence across all of the strategies covered in the training, which in itself is unlikely to be educationally significant, but in conjunction with the improvements in objective competence provides some reassurance of broader skill improvement. Also by directing the participants to undertake problem solving we limited our ability to assess participants' capacity to choose the most appropriate CBT technique. Ideally, further studies are required to determine if social workers, who have received the educational intervention, can competently apply the full suite of cognitive behavioural strategies and make the appropriate choice as to which strategy(s) should be utilised.

It is unknown if the improvements in competence demonstrated in our study translate to in-practice performance, or indeed to improvements in patient outcomes, and these will be important research questions for future studies. The well documented benefits of CBT are largely based on delivery by clinical psychologists, and a brief training intervention is not going to deliver the same specialised depth of understanding and skill. It remains unknown whether brief training will provide social workers with the necessary ability to consistently apply cognitive behavioural strategies over a period of time, and to skilfully make clinical judgements as to the most appropriate technique for each patient. An important element that is missing from the training in this study is ongoing supervision, consultation and feedback for improving the application and retention of clinical skills [39]. Without the use of these methods the skills taught in this intensive education intervention may decay over time.

Given the widespread unmet need for mental health care, it is a high priority for further research to investigate whether a brief educational intervention can teach social workers to competently deliver targeted psychological strategies that will translate to positive outcomes for patients in practice. The large number of social workers expressing interest in participating in the study demonstrates two important points; 1) social workers are interested in receiving training in evidence-based clinical mental health skills for working with common mental health problems, and 2) it is feasible to recruit sufficient numbers of social workers for mental health training and research.

Whilst the focus of this study has been on Australian social work professionals the findings are of broader international interest to other health workers. Social workers do have a strong element of psychosocial care in their practice yet in Australia they receive minimal training in evidence-based clinical psychological skills. Interestingly, the findings of this study are consistent with the significant gains in objective competence observed in a pre-test post-test pilot study [40] of a brief 10-day CBT training intervention conducted in the UK with graduate mental health workers and allied health professionals.

In a similar way to general practitioners, social workers are largely generalist professionals who work on the front line of health and community service provision. They are asked to perform a wide range of tasks with a wide range of client groups which in itself is a role of great expertise. Yet specialisation and clinical competencies are an increasingly important paradigm for social work practice as evidenced by the new opportunities for social workers in primary mental health care. This study provides encouragement for future research into Australian social work practice competencies in primary mental health care.

## Conclusions

This study was novel in both the development of a training package for social workers in evidence-based brief psychological interventions, and the measurement of social worker competence in delivering cognitive behavioural strategies. Social workers were willing to complete continuing education in cognitive behavioural strategies in the context of being evaluated on their competence. The design of the brief intervention was based on practical skills training using evidence-based educational principles, and it proved an effective way to achieve large immediate improvements in objective and self-perceived competence in cognitive behavioural strategies. This study provides preliminary evidence that a brief educational intervention can train social workers to competently deliver targeted cognitive behavioural strategies for people experiencing common mental health problems. Further research is needed to examine the effect of the training on longer term in-practice performance and, importantly, the ability to achieve positive outcomes for patients.

## Competing interests

The authors declare that they have no competing interests.

## Authors' contributions

GA conceptualised the majority of the training including development of training slides, worksheets and video case examples. He also designed the study outcome measures (including the simulated cases and the survey), coordinated the study implementation, undertook all the analysis and wrote the first draft of this manuscript. GB assisted with training development and provided advice on the educational randomised controlled design. LJ assisted with training design and delivery. RB and RM contributed to the training and analysis of the data. JG and LN contributed expertise on general practice mental health care and health system reforms respectively. All authors contributed to the development of the manuscript. All authors read and approved the final manuscript.

## Pre-publication history

The pre-publication history for this paper can be accessed here:

http://www.biomedcentral.com/1472-6963/10/304/prepub
